# Supplementary figures and images for: Outcomes of decitabine treatment for newly diagnosed acute myeloid leukemia in older adults​
Source: PLoS One. 2020 Aug 6;15(8):e0235503. doi: 10.1371/journal.pone.0235503 (PMC7410295; doi:10.1371/journal.pone.0235503)

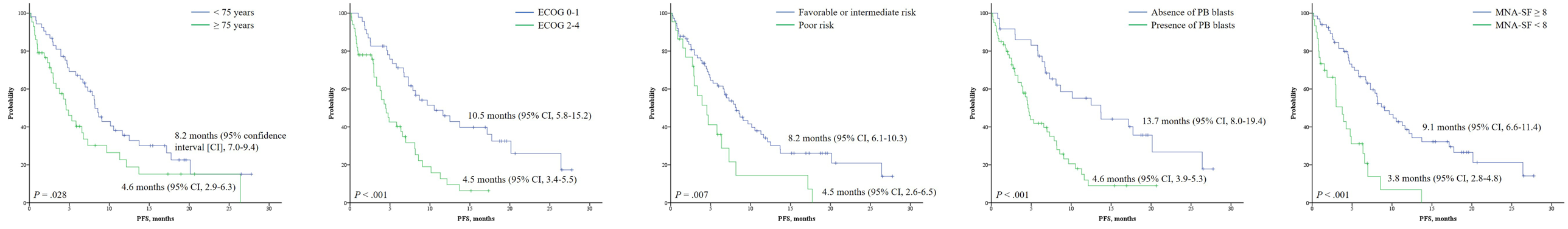

Supplement: S1 Fig — (TIF) [file pone.0235503.s001.tif]

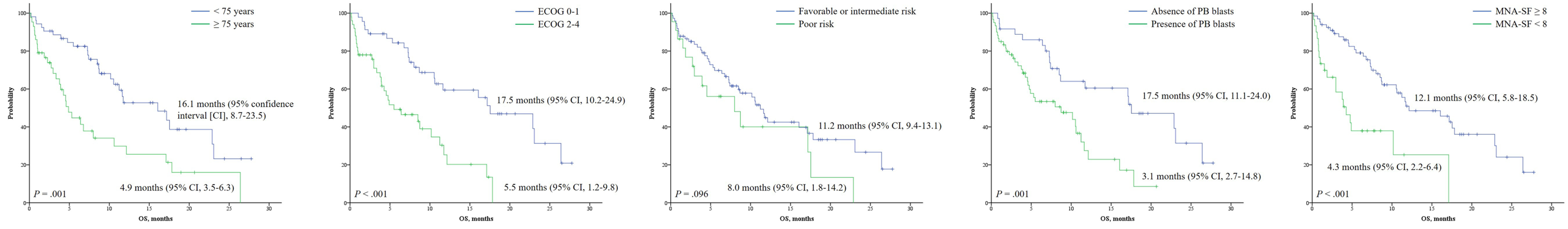

Supplement: S2 Fig — (TIF) [file pone.0235503.s002.tif]
