# Supplementary material for: Outcomes of decitabine treatment for newly diagnosed acute myeloid leukemia in older adults​
Source: PLoS One. 2020 Aug 6;15(8):e0235503. doi: 10.1371/journal.pone.0235503 (PMC7410295; doi:10.1371/journal.pone.0235503)
Supplement: Attachment — Submitted filename: Response to Reviewers.docx [file pone.0235503.s003.docx]

**Reviewer #1:**

Thank you for the opportunity to review this article. This article is well written and deals with an interesting topic on real-life assessment and treatment with decitabine. Subsequently, the authors tried to evaluate prognostic factors.

The major problem is the construction of the article, which oscillates between real-life study and the identification of prognostic factors. This can be seen from the title to the discussion.

The prognostic factor MNA-SF that is put forward is too unreliable in its mode of evaluation. I will focus more on real-life data and secondly on prognostic factors in the minor axis.

TITLE : The major point of the publication is the evaluation of the survival of patients on decitabine in real world and it should be highlighted in the title and not the identification of prognostic factors.

The title should not include prognostic factors.

🡪 We changed the title to “Real-world outcomes of decitabine treatment for newly diagnosed acute myeloid leukemia in older adults”.

Introduction

Indications of decitabine for older adults with AML are still controversial. You must insist on this point the United States Food and Drug Administration did not approve decitabine for the treatment of newly diagnosed AML based on the results of a clinical trial reported by Kantarjian, et al.

The European Medicines Agency Committee for Medicinal Products for Human Use approved decitabine as a first-line treatment for older adults with AML as the Korean Food and Drug Administration (KFDA).

Your objective was to analyze outcomes in real world of decitabine treatment in older patients with newly diagnosed. It should be clearly defined.

Do not put a reference (13) in your objective. The notion of nutritional status should not to be introduced int this part because it is not your main objective.

🡪 We added controversies about the use of decitabine for the first-line treatment of AML. A sentence and a reference about MNA-SF in the part of introduction were removed following the reviewers’ recommendations.

Patients and Methods

How were you able to do the MNA-SF retrospectively? how do you find the data of "Acute illness or psychological stress in the last 3 months" or "Has the patient eaten less in the last 3 months due to lack of appetite, digestive problems, chewing or swallowing difficulties?"

🡪 All institutions participating in this study had acquired all MNA-SF-related indicators at the time of diagnosis of AML, through hospitalization records, baseline nursing records, and nutritional records by on-site nutritionists. We could access the data about "Acute illness and psychological stress in the last 3 months" in hospitalization records and baseline nursing records, and the data about "Has the patient eaten less in the last 3 months due to lack of appetite, digestive problems, chewing or swallowing difficulties?" in baseline nursing records, and nutritional records.

Give more tell about the factors that we included in multivariate analysis. Please detail the multivariate statistical analyses and explain cut-off of MNA-SF.

Most of time : Baseline bone marrow blast, Baseline platelets and / WBC were included in multivariate analysis

🡪 In multivariate analysis by Cox regression model, we used 5 covariates (age, ECOG PS, MNA-SF score, the absence of PB blasts, and cytogenetic risk). Four of them (Age, ECOG PS, MNA-SF score, and the absence of PB blasts) showed significance in univariate analyses of both PFS and OS. Cytogenetic risk (favorable or intermediate risks vs. poor risk) which was statistically associated with improved PFS (*P* = .007), but not OS (*P* = .096), was also added in the model. Baseline bone marrow blasts percentage and platelet count were not associated with survivals in our study population. Therefore, we did not use those factors in multivariate analysis.

Results

I don’t understand this sentence : “Forty-five patients (45/90, 50.0%) showed HI in absolute neutrophil count (29/78, 37.2%)” because you describe 18 patients with HI. I think you should rephrase or add punctuation.

🡪 Those 18 patients did not achieve an objective response (CR or PR), but they showed hematologic improvements (His). We revised the sentences more clearly as following: “18 patients (21.2%) **who did not achieve an objective response** demonstrated hematologic improvement (HI) in PB. Thus, the clinical benefit rate (CR + CRi + PR + HI only) was 52.9% (45/85). **Regardless of achieving an objective response,** 45 patients (45/90, 50.0%) showed HI in absolute neutrophil count (29/78, 37.2%), hemoglobin (32/78, 41.0%) and/or platelet count (28/77, 36.4%).”

Explain in materiel and methods your definition of “infectious complications”. It included bacterial

infections and fungal infection complications?

🡪 We added a sentence, “Infectious complications included any bacterial, viral, fungal, and miscellaneous infection such as Pneumocystis jiroveci during decitabine treatment.” in the Patients and Methods section.

Discussion

The most limiting point is the identification of the MNA-SF, which is unreliable. Its identification as a prognostic factor must be taken with caution.

🡪 We accepted your suggestions and emphasized the limitations of the use of MNA-SF by retrospective measures. We also mentioned the need for prospective studies for confirming the value of MNA-SF in elderly AML.

You do not enough compare your results (cut-offs, results) with other existing publications (10-12) and I added new references:

“Decitabine as a First-Line Treatment for Older Adults Newly Diagnosed with Acute Myeloid Leukemia” Hyunsung Park et al.

“Decitabine improves outcomes in older patients with acute myeloid leukemia and higher blast counts“ Kadia et al.

“Multivariate and subgroup analyses of a randomized, multinational, phase 3 trial of decitabine vs

treatment choice of supportive care or cytarabine in older patients with newly diagnosed acute myeloid leukemia and poor- or intermediate-risk cytogenetics.” Mayer J et al.

🡪 Many thanks for the list of existing publications. We added a comparison of survival and cut-offs and reason for including PB blasts in multivariate analysis rather than BM blasts in the part of discussion.

Figures

Poor quality figures due to a pixel problem, impossible to read

🡪 The resolution of original figures satisfies the guidelines. The Supplement figures shown in the PDF are too small, so it is difficult to see.

You can download the original files from the link at the top right of figure pages in the PDF.

**Reviewer #2:**

1. The authors stated in the “treatment and evaluation paragraph” that “considering the palliative nature of the HMA treatment, disease evaluation was often not performed unless clear signs of disease progression”.

I think that treatment with HMA is not a palliative treatment but an active therapy for elderly AML that can be performed when the patient is considered unfit for aggressive therapy; Unfit patient must be defined based on a multidimensional geriatric assessment that distinguish them clearly from a frail patient for which best supportive care is suggested.

🡪 We intended “palliative nature” as a meaning of different mechanism and responses from traditional intensive chemotherapy. As the reviewer pointed out, the meaning of “palliative nature” is unclear, so we corrected this phrase as follows: “considering the mechanism of action of decitabine and delayed responses different from intensive chemotherapy,”

In absence of a systematic disease evaluation for all patient population with bone marrow aspirate/biopsy how could you determine properly disease response ( es CR/CRi)?

🡪 Patients with new presence of leukemic blasts in peripheral blood or lack/loss of hematologic improvement during treatment were thought of disease progression or no response. In that cases, bone marrow (BM) biopsy were not needed to confirm CR or PR. This study was a retrospective study, and it reflected a real-world practice. However, every patients with CR or PR in our study were confirmed by BM biopsy.

In Table 1 which describes the patients characteristics their multidimensional geriatric assessment is missing. Thus how did you decided that patients were unfit for aggressive chemotherapy or frail? I do not think that ECOG performance status assessment in sufficient for a correct fitness evaluation

🡪 This study is a retrospective study from multicenter. Thus, a uniform multidimensional geriatric assessment was not performed, and physicians of each center individually decided to start decitabine treatment. This might reflect the actual situation of our clinical practice.

2. The highlight and the novelty of the study is that MNA-SF assessment ≥8 predicts improved survival in decitabine treated AML patients.

I think that it should be specified when MNA-SF score was performed. If was performed at diagnosis and/or every how many months during decitabine treatment.

In fact a dynamic assessment is more significative because the score value can change a lot along treatment and on the base of disease response to therapy

🡪 All the MNA-SF-related indicators were collected only once at the time of diagnosis of AML. We absolutely agreed with the reviewer’s point-out, and we discussed more about necessity of a dynamic assessment of MNA-SF, by prospective approach.
