# Supplementary material for: Outcomes of decitabine treatment for newly diagnosed acute myeloid leukemia in older adults​
Source: PLoS One. 2020 Aug 6;15(8):e0235503. doi: 10.1371/journal.pone.0235503 (PMC7410295; doi:10.1371/journal.pone.0235503)
Supplement: Attachment — Submitted filename: Response to Reviewers.docx [file pone.0235503.s004.docx]

Reviewer #1: Article that has improved since the last time, but there are still points that need to be clarified.
Materiels and Methods :
Change title
Definition of real word : Real world data (RWD) in medicine is data derived from a number of sources that are associated with outcomes in a heterogeneous patient population in real-world settings, such as patient surveys, clinical trials, and observational cohort studies.[1] Real-world data refer to observational data as opposed to data gathered in an experimental setting such as a randomized controlled trial (RCT).
Remove real word in the text

🡪 We removed “real world” in the title and introduction section.

I am very dubious about the retrospective evaluation of the MNA-SF, which for me will inevitably be subjective due to its retrospective character and therefore unreliable. You say that the people have partly made a prospective and retrospective evaluation, so why didn't you do the questionnaire from the beginning?

🡪 MNA-SF-related factors were obtained by physicians, nurses, and nutritionists in participating institutions. They were recorded on hospitalization records, baseline nursing records, and nutritional records for purposes unrelated to this study. As reviewers pointed out, MNA-SF scores were retrospectively calculated by reviewing medical records, therefore the reliability of MNA-SF score is inevitably lower than that of prospective studies. Since the predictive power of this score was expected to be weak, it was only described as an additional content according to recommendations. We also described this limitation in discussion section.

You said that the classification was done according to the 2016 classification, but what about before 2016? Did you reclassify the AML?

🡪 AML was initially categorized by WHO classification of myeloid neoplasms and acute leukemia at the time of diagnosis in each patient (i.e., either version 2008 or 2016). We reclassified the category according to the 2016 revision of WHO classification when we obtained and analyzed these data.

The detail of the univariate/multivariate analysis are missing in methods: which criteria, which p...

🡪 We added detailed description about the methods of analysis in statistical analysis section.

PFS and OS should be described in statistical analysis.

🡪 Definition of PFS and OS was moved to statistical analysis section from treatment and evaluation section.

Results
The description of the results on prognostic factors, the most important one, is confusing:
for example: "age, PS, absence of PB... were associated to prolonged OS"
Does it involve univariate, multivariate analysis?

🡪 As a reviewer pointed out, methods of statistics were unclear. We added a clear explanation in methods section and tailed additional markers (univariate/multivariate) in the sentences of result section.

Figures: p and months are missing

🡪 Figure 1A and 1B: Survival duration (months) and 95% CI were already presented in Figure legends.

🡪 Figure S1 and S2: *P*-value was given in lower left corner of each graph. We added survival duration (months) and 95% CI of each curve.

Tables: HR and 95%CI are missing in univariate analysis

🡪 Univariate analysis of survival was conducted by using log-rank test. Thus, univariate P was presented in Table 4, but hazard ratio cannot be calculated in univariate analysis.

Discussion
Difficult in the discussion to see the original points of the article with numbers digits of the publication appearing.

🡪 We expressed the exact number of survivals from other studies more clearly (reference 9-12, 18) to compare with the results of ours.

In a recent publication (Fili et al. 2019, leukemia research) found : The ORR in real-world setting was 33% and median OS in responders was 22.6 months. Which is more than you, how can you explain the difference?

🡪 In the publication (Fili et al. 2019, leukemia research), the ORR was 42% and median OS was 12.7 months in patients treated with first line decitabine (n = 75). These numbers seemed better than our results, but they involved more proportion of better performance status (ECOG PS 1-2 was 88%) than ours. We added explanation about this in the first paragraph of discussion section.
